# Antiseptic Surface Based on Antibacterial Polyethylene Composites with Silver Fillers: Stability in Aqueous Solution

**DOI:** 10.3390/polym16223154

**Published:** 2024-11-13

**Authors:** Marisol Gallegos-García, Zoe V. Quiñones-Jurado, María Azucena González-Lozano, Patricia Ponce-Peña, Miguel Ángel Escobedo-Bretado, G. Cadenas-Pliego, C. Cabello-Alvarado

**Affiliations:** 1 Facultad de Ingeniería, Universidad Autónoma de San Luis Potosí, Av. Dr. Manuel Nava No. 8, San Luis Potosí 78290, San Luis Potosí, Mexico; marisol.gallegos@uaslp.mx; 2 Facultad de Ciencias Químicas, Universidad Juárez del Estado de Durango, Av. Veterinaria S/N, Circuito Universitario, Durango 34120, Durango, Mexico; azucena.gonzalez@ujed.mx (M.A.G.-L.); pponce@ujed.mx (P.P.-P.); miguel.escobedo@ujed.mx (M.Á.E.-B.); 3 Centro de Investigación en Química Aplicada, Saltillo 25315, Coahuila, Mexico; christian.cabello@ciqa.edu.mx

**Keywords:** clean surfaces, silver-polymer, micro-composite, nano-composite, erosion, aqueous

## Abstract

One method to reduce the spread of pathogens is to use clean surfaces. These have long-acting components, and their use would reduce the massive consumption of disinfectants and cleaning products. In order to ensure the safety of these surfaces in water-based systems and prevent mishandling and potential health and environmental risks, this study analyzed the stability of clean surfaces made of polyethylene with three silver compounds with different water solubility. The surfaces were subjected to erosion at 40 °C by immersing them in aqueous solutions of 3% acetic acid (*w*/*v*), 50% ethanol (*v*/*v*), and deionized water. The ionic silver release was monitored in real-time in situ via voltammetry using an Ag/S^2−^ electrode. Analytical methods such as Scanning Electron Microscopy (SEM) and Energy Dispersive X-ray Spectroscopy (EDS) were employed to elucidate the surface alteration. The plastic residue after immersion varied depending on the pH and the type of solvent used, with a higher plastic migration observed when in contact with the water-ethanol mixture. Furthermore, a correlation was identified between surface stability, oxygen composition in the antibacterial, and water solubility, influencing increased surface oxidation.

## 1. Introduction

Preserving a clean surface is crucial for applications in contact with water or moisture, as unchecked proliferation of bacteria, yeasts, or mold can occur [1,2,3,4]. However, the alternative of regular surface disinfection carries the potential risk of excessive chemical consumption, which could harm human health and the environment [5,6], underscoring the need for sustainable aseptic alternatives.

Using durable, clean surfaces has been proposed as a promising and sustainable aseptic alternative. These surfaces have the potential to effectively control microbiological transmissions, prevent biofilm formation, and even deter marine biofouling [7,8,9,10]. This material offers hope for a future where aseptic practices can be more environmentally friendly and sustainable.

Along with other high-level disinfectants, silver (Ag) can be incorporated as metal ions, complexes, or nanoparticles [11,12,13,14,15] in ceramic or polymeric composites to design clean surfaces. Nowadays, Ag-polymer composites are commonly used in textile fibers, surgical tools, and biological devices, offering effective results at low cost [16,17,18,19]. However, the longevity of these antibacterial agents, the stability of the polymeric matrix, and their environmental impact under different conditions need further assessment. In particular, the maximum toxic effect could be magnified in an aqueous medium [20,21,22,23], specifically if these materials are used as containers for aqueous solutions or come in contact with aquatic ecosystems.

Since polyethylene is one of the most widely used plastics and is very suitable for combining melt blending with antimicrobials to create clean surfaces [23], it is interesting to validate the surface stability and the release of antibacterial agents after contact with an aqueous solution.

As for the antibacterial activity of silver, the literature reported that silver ions interact better with bacteria than nanoparticles unless they have good dispersion, their size is within 10–20 nm, and their exposure to bacteria increases. This last factor is how they could provide the full benefits of nanotechnology [24].

This study evaluated three different Ag-polymer composite surfaces. Their ability to release silver depends on their chemical structure. They originate from a polyethylene matrix with silver ions in a soluble glass, silver salt, and silver nanoparticles incorporated.

The degree of immobilization of silver ions depends on the structure of the incorporated antimicrobial additive. For instance, the solubility of silver ions in water will increase when they are integrated as a salt, whereas silver ions embedded into glass will have lower interaction with water and even less for metallic silver nanoparticles [25].

The rigorous method of overall migration was applied to promote accelerated erosion of the antibacterial samples. This method involves subjecting the samples to specific conditions, such as high temperatures and exposure to certain substances, to simulate the effects of long-term use. It is a procedure proved and accepted by agencies such as the Food and Drug Administration (FDA) and the European Food Safety Authority (EFSA), the latter by regulation No (EU)10/2011 [26]. Complementary time in-situ silver ions quantification was conducted with a silver/sulfide ion-selective electrode. Scanning Electron Microscopy (SEM) was used to study the morphology of migrated material after the overall migration test. Energy dispersive X-ray spectroscopy (EDS) was coupled to microscopy to determine its elemental composition. For the characterization of the antibacterial polymeric surface after erosion at an aqueous system, X-ray photoelectron spectrometry (XPS) was performed.

It is crucial to redesign the existing antiseptic surfaces for product recirculation. This is necessary to adapt them to a longer cycle or to limit their use, thereby preventing any collateral effects on health and the environment.

The primary goal of this study is to investigate the effect of antimicrobial release from a clean surface on the durability and integrity of the polymer matrix. The findings could have significant implications, especially when submerging the surface in aqueous solutions.

The interest in validating silver-based antimicrobials is that, upon contact with water, they can present less harmfulness than those generated by other heavy metals such as copper. Silver, in its ionic form without oxidizing agents or complexing substances, can remain completely stable in the Ag^+1^ oxidation state [27]. However, silver in a higher oxidation state (Ag^++^ and Ag^+++^) or in contact with mineral salts and organic substances in an aqueous medium will trend to complex through processes such as ion exchange, adsorption, and chelation.

In addition, if thiol groups exist, they can promote sedimentation [28]. Other reports indicated that silver nanoparticles in hard water can promote their association or agglomeration, reducing their reactivity to that of a micro-size silver particle [29].

Therefore, due to the wide variety of silver chemical species, it is relevant to know their stability before developing new Ag-polymer components for clean surfaces. Incorrect management of these components could lead to the unintended release of silver into the environment, potentially contributing to the high incidence of Ag-micro and nano-particles in wastewater treatment plants [30].

## 2. Materials and Methods

### 2.1. Clean Surface Production

Clean surface samples were produced using a cast film extrusion process in a Killion single-screw extruder with L/D of 24:1, model KTS-100 (Soler & Palau Inc., Montville, NJ, USA), coupled to a die for a single-layer film of 25 µm. The extruder and die sections were set at 170 °C and 190 °C, respectively, with an extruder speed of 300 rpm. The linear low-density polyethylene (LLDPE) plastic matrix was provided by Marubeni America Corporation (New York, NY, USA), and it contained 2000 mg/kg of different antibacterial additives. These additives were all silver-based and had solubility dependent on their chemical structure. Sample description is presented in Table 1, where one comprises a composition of silver salt (Ag Salt), the other contains silver ions integrated into a soluble glass (Ag-Soluble Glass), and the third antibacterial plastic is based on the incorporation of nanoparticles of both silver and zinc oxide (Ag·ZnO NPs).

### 2.2. Separation of Antibacterial Filler from Plastic by Calcination

The raw materials of the silver-polymer compounds (Ag Salt, Ag-Soluble Glass, and Ag·ZnO NPs) were separated from the plastic matrix. This process involved the removal of the LLDPE polymer (Linear low-density polyethylene) by calcination at 800 °C for 30 min using a Thermo Scientific Thermolyne 1300 electric furnace, with a capacity of 2.2 L and a maximum temperature control of 1200 °C (Thermolyne Furnace Benchtop Industrial, central Waltham, MA, USA).

### 2.3. Migration of Non-Volatile from Antibacterial Plastics After Immersion in Aqueous Solutions

The migration of substances from the antibacterial surface was rigorously tested according to the standard for plastics outlined in the Official Journal of the European Union (EU) No. 10/2011) [26]. This method measures the amount of plastic monomers or additives that migrate from plastic when exposed to a specific temperature and aqueous solution. The migration from antibacterial plastic samples was measured, each approximately 0.0025 × 2.5 × 10 cm in size. The surfaces were exposed to solutions for erosion, a crucial step that simulates real-life conditions; these consisted of 3% acetic acid (*m*/*v*) and 50% ethanol (*v*/*v*), respectively. Deionized water was used as a reference. The immersion was for 10 days, maintaining the temperature at 40 °C; this last condition promotes accelerated erosion, which, according to the standard, corresponds to 30 days at room temperature. The samples were removed from the solution, evaporating the remaining solution. The weight of the residual substances was determined [26]. Each experiment was conducted with four samples for each of the aqueous simulants.

### 2.4. Silver Releasing from Antibacterial Plastics Immersed in Aqueous Solutions

Over ten days, it measured the released ionic silver (Ag^+^ ions) using a potentiometer equipped with a silver/sulfide selective ion electrode (Ag/S^2−^), (Cole Parmer, Vernon Hills, IL, USA). Silver quantification was obtained by correlating the detected voltage to a calibration curve at 0.01, 0.05, 0.1, 1.0, and 10 ppm of silver.

### 2.5. EDS-Scanning Electron Microscopy for Characterization of Antibacterial Filler and Migrated Residue

The shape and composition of the bare material of the antimicrobial fillers, obtained after the calcination of the plastic matrix, were analyzed. This analysis was crucial to understanding the properties of the fillers and their potential antibacterial effects. These remnants included the residues deposited at the bottom of the glass vessel following the evaporation of the solution that facilitated the erosion of the antiseptic surface formed by each silver-polymer composite. The analysis was conducted using Transmission Electron Microscopy-Energy Dispersive Spectroscopy (TEM-EDS) (JEM-1230, JEOL Inc., Tokyo, Japan), which operated at 100 kV in bright field imaging mode. Scanning Electron Microscopy-Energy Dispersive Spectroscopy (SEM-EDS) was also performed using a Philips FEI microscope model XL-30-PW6630/01 operated at 20 kV (Philips, Portland, OR, USA).

### 2.6. Determination of Migrated Residue Obtained After Evaporating the Remaining Solution from Antibacterial Plastic

After the solutions used to erode the antibacterial plastic were removed by evaporation, digital photographs of the migrated residue were taken. The image corresponds to the bottom of the glass tube with a diameter of 3.5 cm. A Moto G7 EE smartphone (Motorola, Schaumburg, IL, USA) was used, focusing 30 cm from the base of the test tube.

### 2.7. XPS for Characterization of the Antibacterial Polymeric Surface After Erosion at an Aqueous System

The X-ray photoelectron spectroscopy (XPS) study was realized in the K-ALPHA spectrophotometer with an accelerating voltage of 5–25 keV (Thermo Scientific, model XL-30 Phillips, Waltham, MA, USA). This instrument has a monochromatic X-ray source with a binding energy of 0–1350 eV and a depth of 400 μm. The samples were not pre-treated.

## 3. Results and Discussion

### 3.1. Characterization of Antibacterial Additive

**Figure 1:**
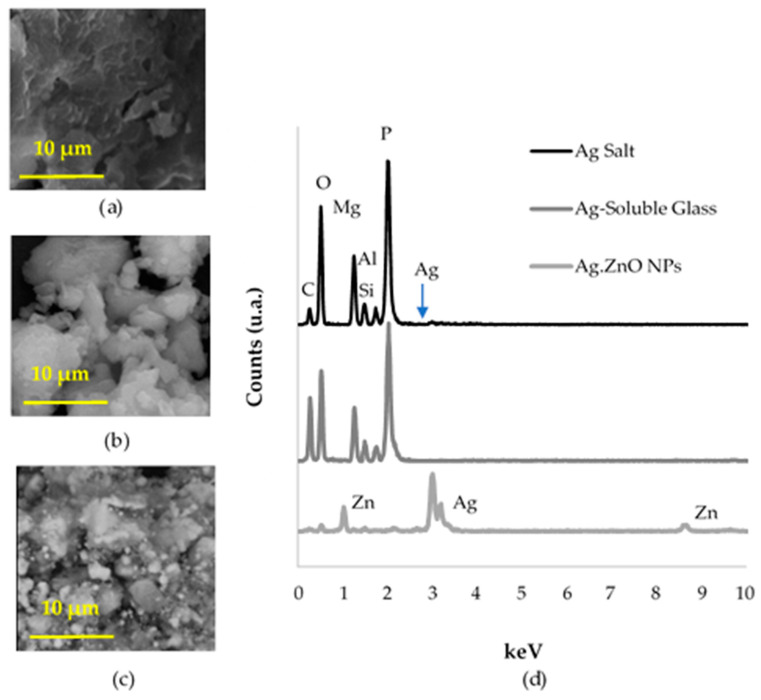
Morphology and elemental chemical analysis of the antibacterial resulting from the calcination of plastic. (**a**) Ag Salt, (**b**) Ag-Soluble Glass, (**c**) Ag·ZnO NPs, and (**d**) EDS spectra.

For the chemical identification of antibacterial additives, they were separated from the plastic by thermal degradation of the polymer. Figure 1a–c show the morphology and Figure 1d and Table 2 show the EDS spectra and the elemental composition of the material resulting from calcination of antibacterial plastic, respectively. Figure 1d compares the EDS spectra for each antibacterial additive, where a similar composition for Ag Salt and Ag-Soluble Glass was identified (Table 2). There, phosphorus, magnesium, and oxygen are the main constituent elements, and characteristic peaks of aluminum and silicon are also present. Despite this, the silver element was barely perceptible for Ag Salt composition, and no silver was detected for Ag-Soluble Glass (Figure 1d). The antibacterial additive composition (Ag·ZnO NPs) was confirmed by the presence of silver, zinc, oxygen, magnesium, and aluminum (Table 2). After the plastic was burned at 800 °C, the resulting Ag Salt additive appeared as a compacted material, indicating an incipient melting process (Figure 1a). Even though Ag-Soluble Glass and Ag Salt have similar compositions (see Table 2), there are differences in their morphology. The glass shows particles with defined edges, indicating no signs of melting (refer to Figure 1b). On the other hand, the Ag·ZnO NPs material was observed as fine particle agglomerates (see Figure 1c).

### 3.2. Erosion of Antibacterial Plastics by Aqueous Solvents

Overall migration method determined the stability of the polymeric materials through extraction with food-simulants solvents. As shown in Table 3, the extraction of a residue for each Ag-polymer composite and nanocomposite in all the liquid environments was observed. The residue obtained from plastics after immersion for ten days depended on the pH condition of each aqueous solution. The amount migrated was slightly higher in the case of antibacterial plastics immersed in the ethanol solution. The latter imparted a condition of pH 4.4 and a material extraction capability between 10.6 to 11.4 mg/dm^2^. In the trial with the acetic acid solution at pH 2.25, the extracted weight was 6.4 to 8.5 mg/dm^2^, while in distilled water at neutral pH, the extracted weight was 4.7 to 6.1 mg/dm^2^. These results underscore the need for further research on the stability of polymeric materials in different solvents, highlighting the urgency of this topic.

The alcoholic solution demonstrated the highest effectiveness in extracting residue (50% *v*/*v*), consistent with findings from other studies [31]. This significant extraction rate is related to the swelling of the polyethylene, a phenomenon observed in similar experiments.

Based on the standard for plastics of the Official Journal of the European Union [26], which ensures the safety of plastics in contact with liquid foodstuffs, it was confirmed that using the Ag-polymer composites in the acetic acid solution and deionized water is appropriate. However, the inertness of the Ag-polymer composites with the alcoholic solution is not guaranteed, as the amount of residue was higher than the permitted limit of 10 mg/dm^2^ [26]. Therefore, Ag-polymer composites with a polyethylene matrix may not be suitable for applications that involve alcohol immersion, such as medical ones, because they may have a greater incidence in the release of antibacterial and monomers liberation; the latter is currently a cause of endocrine disruption in mammals [32].

### 3.3. Characterization of Migrated Residue from the Antibacterial Plastic

**Figure 2:**
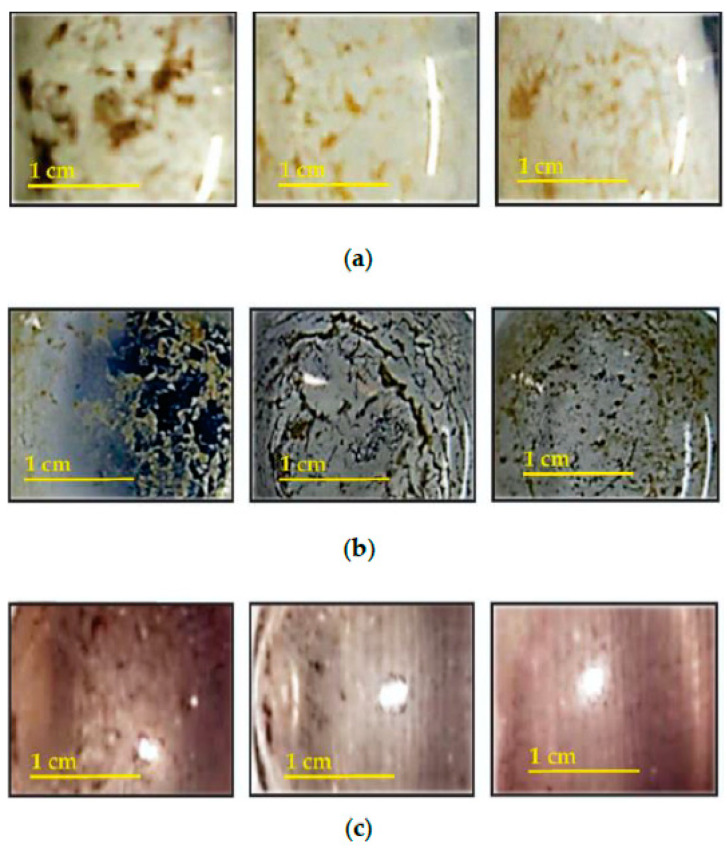
Photographs of the migrated residue from antibacterial plastic immersed in (**a**) ethanol solution, (**b**) Acetic acid solution, and (**c**) deionized water. The first column corresponds to the residue from Ag salt, the second to 2 Ag-Soluble Glass, and the third to Ag·ZnO NPs antibacterial plastic.

The material migrated from the antibacterial plastics by the extraction test using a solution of deionized water (c), acetic acid solution (b), and ethanol (a) is shown in Figure 2. When tested in deionized water, a small amount of residue was observed (Figure 2c). However, when tested with acetic acid and ethanol solution, a visible amount of residues of different colors were observed for each antibacterial plastic. In Figure 2a, the residues that originated from the ethanol solution appeared predominantly like wax in sepia tones. Due to the waxy appearance, it is presumed that most of the migrated components belonged to the plastic matrix (monomers or oligomers), which would accelerate the erosion of the plastic. The effect of erosion produced by ethanol in the polymeric matrix is consistent with a study by Jiangfang, Z. et al., which demonstrated similar results by the swelling caused in the polymer [31].

**Figure 3:**
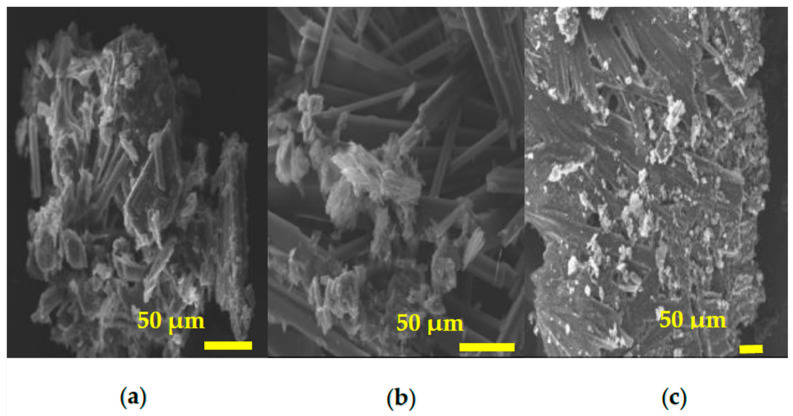
Micrographs of residue migrated from antibacterial plastic, obtained after immersion in Acetic acid solution, (**a**) Ag salt, (**b**) Ag-Soluble Glass, and (**c**) Ag·ZnO Nps.

On the other hand, the material migrated from antibacterial plastics in an acetic acid solution could be considered to be more similar to residues of metallic salt due to its darker color (Figure 2b). These residues were analyzed by SEM and EDS, which detected two different phases. The largest phase exhibited elongated crystals in an acicular shape, while the other seemed like irregular agglomerates (Figure 3). The elongated crystals comprised chlorine and potassium, while the other phase contained carbon and silicon, as indicated in Table 4. Despite the higher acidity condition imparted by the 3% acetic acid solution, which could leach a higher concentration of inorganic components, this analysis method did not detect the silver element. Therefore, we did not observe elements of the antimicrobial additive. However, in irregular agglomerates, components from the plastic matrix and others commonly used to manufacture plastic products were detected, such as anti-blocking and slip agents based on silicon [33]. Thus, through acetic solution treatment, it was confirmed that erosion of the plastic matrix is possible in the different solutions and that the inorganic antibacterial components remain stable, fulfilling the durability attribute.

### 3.4. Silver Concentration Released from the Antibacterial Plastic

**Figure 4:**
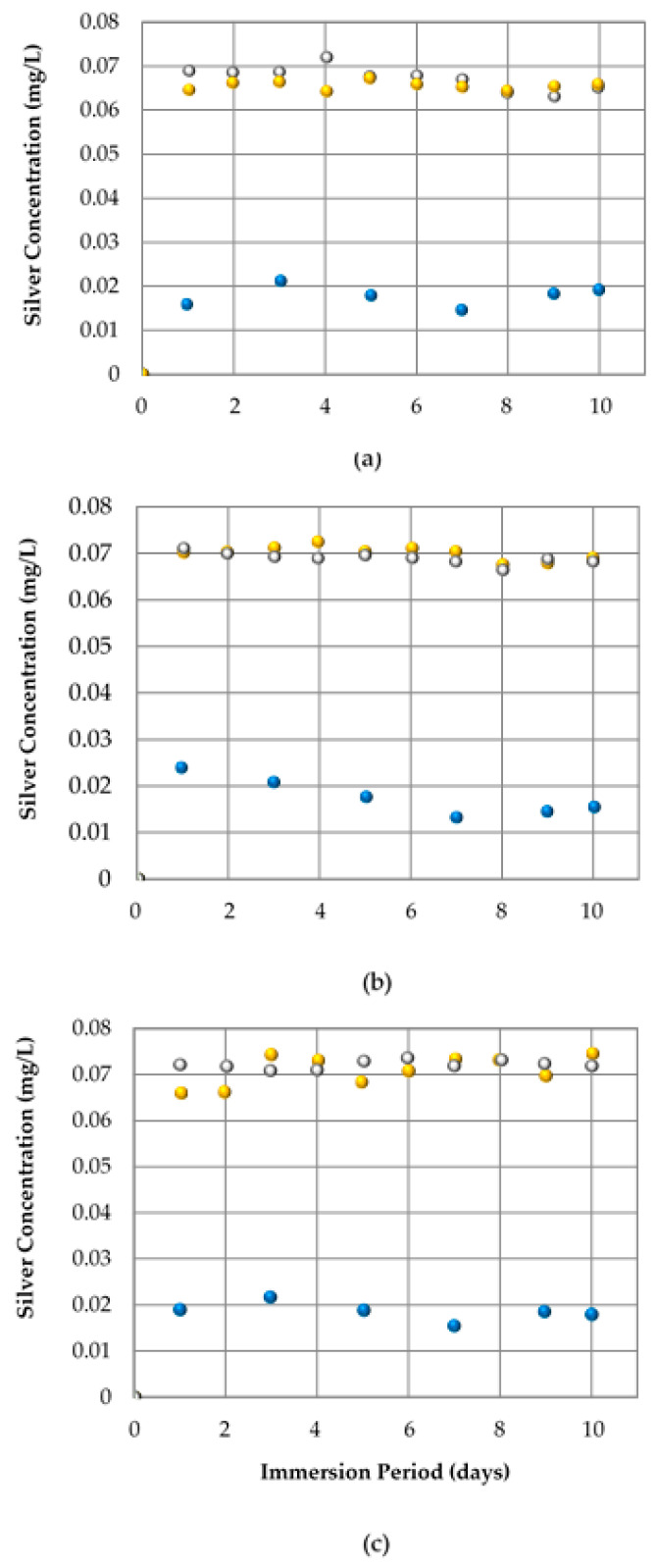
Release kinetics of ionic silver from Ag Salt Plastic (**a**), Ag-Soluble Glass Plastic (**b**), and Ag·ZnO NPs Plastic (**c**) at contact with: deionized water in blue, acetic acid solution in grey, and ethanol solution in yellow.

Complementary to, and at the same time as, the overall migration method, the Ag^+^ ions concentration was detected in situ using the Ag/S^2−^ electrode. Figure 4 shows silver ion release kinetics for the three antibacterial plastics under immersion for ten days. The silver concentration was between 0.065 and 0.075 mg/L for the acidic condition and close to 0.02 mg/L for the neutral pH, in consequence in both pH conditions, the antibacterial plastic released a concentration lower than that restricted for human consumption, reported at 0.1 mg Ag/L, which indicates the stability of silver in the polymeric matrix [34]. Under acidic conditions, a trend in the ionic silver extraction was observed for the different types of clean surfaces of Ag-polymer composites. The amount of silver released was the lowest in the Ag salt (Figure 4a) and the highest with a slight increase in silver release for the Ag·ZnO nano-composite (Figure 4c), which can be related to the concentration of silver element in the different antibacterial plastics, as is shown in Table 2. Meanwhile, in the case of both Ag polymers with about 1 wt% silver content (Table 2), the Ag-Soluble Glass (Figure 4b) showed a slight increase in silver release compared to the Ag salt. Finally, in this trial, it was also seen that at pH 2.25 of the acetic acid solution, a similar silver concentration than that at pH 4.4 by the ethanol solution was extracted, even though the latter condition solubilized the polymer matrix.

### 3.5. Antibacterial Polymeric Surface After Erosion at an Aqueous System

**Figure 5:**
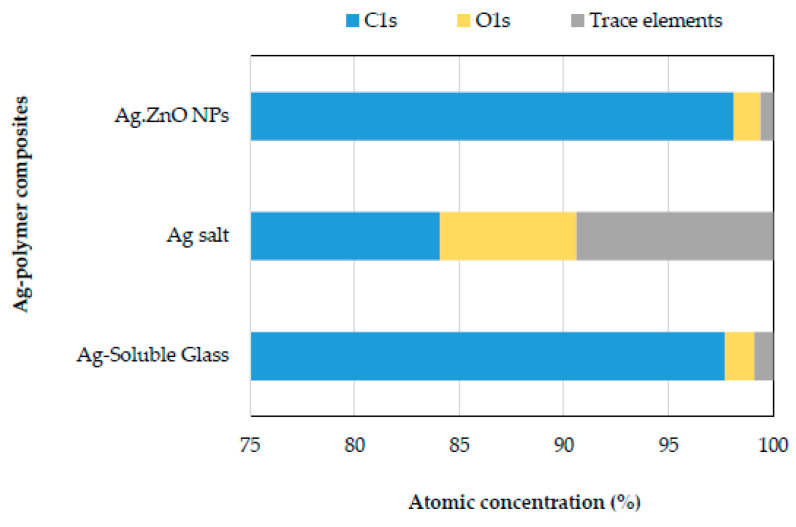
Atomic percentage of antibacterial Plastics surface composition after immersion in Acetic acid solution.

After identifying that the higher the acidity, the higher the leaching of silver ions from the antimicrobial plastic compounds, and excluding the condition of accelerated erosion with the use of acidic ethanol solution due to the polymeric matrix extraction, the presence of chemical elements residing on the surface after attack with acetic solution were evaluated. Figure 5 shows the comparison of detected elements from the XPS spectra of the different plastic substrates; the predominant atomic percentage was for the C1s in all samples, which corresponds to the LLDPE polymer matrix, followed by the oxygen signal for O1s and then as a trace element silicon from the Si2p signal. At a lower level, other trace elements were observed as the signals Cl2p, Ca2p, N1s, Na1s for the antibacterial plastic with Ag salt and Zn2p, Cu2p3 for the Ag·ZnO NPs. Comparing the atomic percentage of the oxygen element, we can see that the substrates of Ag·ZnO NPs and Ag-Soluble Glass presented a similar level of oxygen (<2%), and only the Ag salt plastic showed a higher oxygen content on the polymer surface, with an atomic percentage content of 6.5% (Figure 5). This suggests that the higher solubility of antibacterial additive, the higher impact on its leaching and, consequently, on the oxidative level of the polymer. As for the oxygen analysis of the antibacterial additive (Table 2), the Ag·ZnO NPs presented only 15.66 wt.%, while the Ag Salt and Ag-Soluble Glass approached 50 wt.%; thus, a low oxygen content of the antibacterial additive has little influence on the degree of oxidation of the polymer after contact with an aqueous system. Also, the surface of the plastic containing Ag-Soluble Glass, despite its high oxygen concentration, as presented by the Ag Salt, the atomic percentage of O1s developed on the surface of the polymer was similar to that caused by the additive Ag·ZnO NPs. Therefore, the concentration of the oxygen component of antimicrobial additive and the degree of water solubility will be a conditioning factor in causing oxidative erosion of clean polymer surfaces during their use in contact with aqueous systems. Therefore, it could be considered that the use of antimicrobial salts or other antimicrobial compounds highly soluble in water or easily migrated from the clean surface upon contact with water would not be recommended because of the short performance they would represent, and because they could imply that the higher surface level of oxidation would more easily erode the surface.

## 4. Conclusions

In this study, we confirmed the release of silver ions from antiseptic surfaces made of Ag·ZnO NPs, Ag soluble glass, or Ag salt after exposure to water. This release indicates the potential for these antiseptic plastics to provide antibacterial control, at least to create a bacteriostatic surface. Although the primary objective of this research was not to demonstrate the effectiveness of reducing pathogen spread on the tested surfaces or to quantify biological activity against different bacteria, it served as a foundation for understanding how the components and solubility of antibacterial fillers influence the erosion of polyethylene plastic surfaces when exposed to water. We also compared the interaction with aqueous solutions containing 3% (*m*/*v*) acetic acid and 50% (*v*/*v*) ethanol to achieve an accelerated degradation. Therefore, it is crucial to know the stability of antiseptic plastics and limit their use to the appropriate conditions to achieve the desired effect without affecting health or the environment. The clean surfaces formed with nano- and micro-silver composites in a polyolefin-based polymeric matrix such as LLDPE demonstrated high stability in contact with moisture. The low release of silver in ionic form and the very low probability of particle detachment of antimicrobial were critical factors in this stability, underlining the potential for their use in aqueous systems. The restriction detected for this type of material was due to its contact with alcohol solutions, which could lead to increased migration of the polymeric matrix. The release of silver ions from these antimicrobials showed high stability and controlled dosage, adhering to safe limits for human use in containers or water reservoirs. These antimicrobials can also be designed for antiseptic surfaces in highly acidic conditions, such as pH 2.25, as the release of silver ions remains controlled. The use of Ag·ZnO NPs nanocomposite and Ag soluble glass as antimicrobial additives in creating a clean surface with LLDPE showed the best performance in terms of polymer matrix stability, and its silver ion release was comparable to that of Ag salt plastics. Besides extending the antibacterial performance, it is possible to reduce aging and plastic degradation, which is very important to consider for producing antibacterial clean surfaces intended for applications under wet conditions. Finally, antimicrobial additives with low oxygen content are preferred, as this also impacts promoting oxidation of the polymer matrix. In conclusion, this small contribution aims to encourage the appropriate use of materials to address pressing needs, such as safe water storage, which can serve as an alternative solution to water scarcity. Due to the wide variety of silver chemical species, it is relevant to know their stability before developing new Ag-polymer components for clean surfaces. Incorrect management of these components could lead to the unintended release of silver into the environment, potentially contributing to the high incidence of Ag-micro and nanoparticles in bodies of water or plastic tanks for water deposit. One proposal is antiseptic surface tanks that can be manufactured to collect rainwater. By promoting innovative materials, we hope to contribute to the global efforts to combat water scarcity.

## Figures and Tables

**Table 1:** Identification of Antibacterial Plastic.

Silver-Polymer Composite	Antibacterial Additive	Level of Insolubility
Micro-composite	Ag Salt	High
	Ag-Soluble Glass	Very high
Nano-composite	Ag·ZnO NPs	Extreme

**Table 2:** Chemical composition of Silver compounds contained in the antibacterial plastic.

Element	Ag Salt wt%	Ag-Soluble Glass wt%	Ag·ZnO NPs wt%
O K	51.01	49.55	15.66
MgK	12.23	12.22	1.50
AlK	3.66	3.78	1.67
SiK	2.27	3.00	0
P K	29.52	30.45	0
AgL	1.31	1.00	65.79
ZnK	0	0	15.38

**Table 3:** Migration behavior of antibacterial plastics by extraction with aqueous solvents.

	Non-Volatile Substances (mg/dm^2^)		
Antibacterial Plastics	Acetic Acid Solution (pH = 2.25)	Ethanol Solution (pH = 4.4)	Deionized Water (pH = 7.0)
Ag·ZnO NPs	8.5	11.4	6.1
Ag-Soluble Glass	6.4	11.3	4.8
Ag salt	7.4	10.6	4.7

**Table 4:** Chemical elements of the phases detected into residue migrated after immersion in acid simulant.

Acicular Crystals		Irregular Agglomerates	
Element	Weight %	Element	Weight %
Cl	49.36	C	64.27
K	50.64	O	32.39
-	-	Si	3.34

## Data Availability

The original contributions presented in the study are included in the article, further inquiries can be directed to the corresponding author.
